# BCG in the fight against cancer: exploring its applications in diverse tumour types and future directions

**DOI:** 10.3332/ecancer.2026.2142

**Published:** 2026-06-09

**Authors:** P Ignacio A Bianco, O Jeismar M Carballo, P Isaac R Blanca, Ana Federica Convit

**Affiliations:** 1 Jacinto Convit World Organization Inc., Pompano Beach, FL 33069, USA; 2 University of Bologna, 40126 Bologna, Italy; 3 Unidad Experimental de Inmunoterapia, Fundación Jacinto Convit, Caracas 1071, Venezuela

**Keywords:** Bacillus Calmette Guérin vaccine, urinary bladder neoplasms, immunotherapy, trained immunity, intralesional injections, cancer vaccines

## Abstract

Bacillus Calmette–Guérin (BCG), originally a tuberculosis vaccine, is a standard immunotherapy for non-muscle-invasive bladder cancer (NMIBC). This review summarises BCG biology, including trained immunity, and evaluates clinical and translational evidence for BCG across solid tumours. We aim to distinguish guideline-supported indications from investigational uses and identify contexts where BCG may remain clinically relevant or trial-ready. Evidence is strongest in NMIBC, where outcomes depend on induction plus maintenance schedules and appropriate patient selection. Outside the bladder, intralesional and vaccine-adjuvant approaches show signals in selected melanoma and vaccine settings, while historical lung and colorectal trials largely failed to translate into durable benefit. Emerging preclinical work in hepatocellular carcinoma and breast cancer suggests potential synergy with modern immunotherapy strategies. BCG should be framed as a platform immunomodulator whose value beyond NMIBC requires mechanism-guided trials, clear safety boundaries and clinically anchored endpoints.

## Introduction

Bacillus Calmette–Guérin (BCG) was repurposed as intravesical immunotherapy for bladder cancer following its success as a tuberculosis vaccine. Its anti-tumour activity is multifaceted. Directly, BCG induces apoptosis, necrosis and oxidative stress through caspase activation, tumour necrosis factor–alpha (TNF-α) signaling and production of nitric oxide and reactive oxygen species (ROS), damaging tumour DNA and proteins [1]. BCG’s preferential cytotoxicity for tumour over normal urothelium appears to reflect selective uptake: malignant urothelial cells commonly activate Rac1/Cdc42-Pak1-dependent macropinocytosis, enabling BCG entry and concentrating downstream cytotoxic and immune effects in tumour cells [2].

BCG also acts as a pathogen-associated molecular pattern that engages pattern-recognition receptors (notably TLRs) on antigen-presenting cells (APC) and, in some contexts, on tumour cells, driving T helper 1 (Th1)-polarised cytokines (e.g., Interleukin (IL) IL-2/IL-12,‑2/IL‑12, Interferon (IFN) IFN-γ ‑γ and TNF‑α) and recruitment of CD8⁺ T cells, macrophages, natural killer cells and neutrophils. Neutrophils can contribute direct cytotoxicity via TNF-related apoptosis-inducing ligand release and neutrophil extracellular trap formation. Response heterogeneity likely reflects at least three non-mutually exclusive factors: (i) some cancer cells internalise BCG and acquire APC-like cytokine signaling; (ii) BCG can trigger caspase-dependent cytotoxicity with ROS-mediated injury and cell-cycle arrest and (iii) BCG may upregulate PD‑L1 on tumour and inflammatory cells, partially counteracting cell-mediated immunity [3].

BCG’s modern oncologic use was established in bladder cancer, where randomised trials showed intravesical BCG reduced recurrence relative to chemotherapy controls and long-term outcomes improved when induction was followed by maintenance dosing [4, 5]. International guidelines consistently recommend intravesical BCG for appropriately selected intermediate and high-risk non-muscle-invasive bladder cancer (NMIBC) after transurethral resection of bladder tumour, because it reduces recurrence and (in higher-risk groups) delays or reduces progression. For optimal efficacy, guidelines specify that BCG should not be delivered as induction alone: a complete schedule includes a 6-week induction course followed by maintenance instillations (commonly 3-weekly cycles at 3, 6, 12, 18, 24, 30 and 36 months), with duration tailored to risk, toxicity and BCG availability [6, 7]. Taken together, the randomised-trial evidence and guideline-endorsed use of intravesical BCG in NMIBC provide a clinical proof of principle that BCG can act as an effective, controllable, locally delivered immune therapy. This success has prompted efforts to redeploy BCG as an *in situ* immune stimulus in other tumour types.

Importantly, BCG is not a single product but a family of live-attenuated sub-strains with measurable biological and clinical differences. Comparative analyses in NMIBC suggest that strain selection can influence immunogenicity and tolerability, although adequate maintenance remains a dominant driver of durable benefit [8, 9]. These same properties matter when BCG is repurposed as a Th1-biased adjuvant in therapeutic cancer vaccines: more reactogenic preparations can amplify local innate activation and dendritic-cell recruitment, whereas less reactogenic strains may reduce injection-site morbidity and discontinuation risk [10, 11].

Interest in BCG beyond the bladder has resurfaced alongside modern immuno-oncology because BCG can (i) directly injure tumour cells, (ii) activate innate sensors that set up Th1-polarised inflammation and (iii) induce trained immunity that may heighten subsequent antitumour responses. Most contemporary programs still center on bladder cancer, but smaller bodies of evidence exist in melanoma, hepatocellular carcinoma (HCC) and vaccine-adjuvant settings. The sections below focus on where signals are reproducible, where they have failed to translate, and what trial designs could realistically move the field forward. Table 1 summarises the evidence landscape and practical niches for BCG beyond NMIBC.

### BCG in melanoma: intralesional immunotherapy, combination approaches and vaccine-adjuvant strategies

Melanoma remains a primary target for BCG beyond the bladder due to the accessibility of cutaneous metastases. Intralesional therapy exploits this accessibility: in-transit and cutaneous melanoma metastases are one of the few solid-tumour settings where BCG can be delivered directly into tumour deposits. Intralesional BCG triggers a Th1-skewed inflammatory milieu through pattern-recognition receptor signaling and can recruit dendritic cells, macrophages and cytotoxic lymphocytes within injected lesions, providing a plausible *in situ* vaccination effect [12].

### Historical context and early outcomes

Historically, intralesional BCG produced frequent regression of injected lesions and occasional regression of uninjected lesions (abscopal-type effects), but enthusiasm declined due to systemic adverse events when dosing and patient selection were not tightly controlled [12]. In the current era, in which effective systemic therapies exist, intralesional BCG is best viewed as a niche, local-control strategy or a priming maneuver within carefully monitored combinations, not a stand-alone systemic therapy.

### Combination with checkpoint inhibitors

A cautionary example is a Phase I study that administered intralesional BCG followed by ipilimumab; the trial was stopped early after severe immune-mediated colitis/hepatitis occurred without clear clinical benefit. Broadening of autoantibody repertoires tracked with escalating BCG dose and preceded toxicity, supporting autoantibody monitoring as a practical safety biomarker when pairing BCG with checkpoint blockade [13].

### Combination with IL-2

In contrast, pairing intralesional BCG with IL‑2 has shown more consistent clinical activity with tolerable toxicity. In a contemporary series of stage III/IV disease with cutaneous/subcutaneous metastases, the combination achieved high local response rates, including complete responses in many injected lesions, with occasional systemic responses, consistent with synergy between BCG-driven innate priming and IL-2-mediated T-cell expansion [14].

### BCG as a vaccine adjuvant: the CSF-470 experience

BCG’s most persuasive ‘beyond intralesional’ melanoma signal comes from its use as a vaccine adjuvant. In the randomised CASVAC-0401 study, the CSF‑470 (VACCIMEL) allogeneic whole-cell vaccine combined with BCG and granulocyte-macrophage colony-stimulating factor improved distant metastasis-free survival compared with IFN-α2b with acceptable tolerability [15]. Correlative immune analyses showed vaccine-induced T‑cell reactivity against shared melanoma antigens and predicted neoantigens, with functional cytotoxicity and delayed-type hypersensitivity responses that tracked clinical benefit [16].

### Alternative BCG-adjuvanted vaccine strategies

Not all BCG-adjuvanted melanoma vaccines have translated into survival gains. In Canvaxin™ programs, adding BCG did not produce a clear overall survival advantage in broadly selected populations, underscoring that adjuvant potency alone does not overcome weak antigen selection or advanced disease biology [17]. Across autologous and whole-cell vaccine experiences, delayed-type hypersensitivity has repeatedly behaved as an on-treatment pharmacodynamic marker and, in some cohorts, stratified patients with superior long-term outcomes, suggesting that future BCG-adjuvanted vaccines should prospectively use immune readouts to enrich for responders rather than treat unselected populations [18].

### Expanded trials and future directions

Practically, the melanoma lesson is bifurcated: (i) intralesional BCG can deliver high local control (especially with IL‑2) and is worth testing as a priming agent; (ii) as a vaccine adjuvant, BCG can support durable systemic immunity when paired with a credible antigen source (e.g., whole-cell vaccines) and when immune monitoring is built into the trial [14–16]. The main risk is over-activation: checkpoint combinations require conservative dosing, early stopping rules and biomarker-triggered monitoring for immune toxicity [13].

## BCG in lung cancer: intrapleural and vaccine-adjuvant trials show limited, non-reproducible benefit

BCG has been repeatedly tested in lung cancer, mainly as postoperative intrapleural therapy or as a vaccine adjuvant, because most lung tumours are not amenable to intratumoural injection. Small early studies suggested possible benefit in stage I disease, but the best-controlled randomised data in stage I non-small cell lung cancer did not demonstrate a survival or disease-free survival advantage for intrapleural BCG [19, 20]. Vaccine-adjuvant strategies were also largely negative; for example, in limited-stage small-cell lung cancer, adjuvant vaccination with BEC2/BCG did not improve survival, progression or quality of life versus placebo [21]. Taken together, the lung literature reads as a cautionary tale: signals were stage-restricted, inconsistent and not reproducible in rigorous trials. Any renewed lung-cancer interest would therefore need a mechanistically distinct approach rather than repeating intrapleural adjuvant paradigms.

## BCG in colorectal cancer: early postoperative signals, negative cooperative-group trials and a stage II niche for BCG-adjuvanted vaccines

Colorectal cancer was an early testbed for ‘nonspecific’ immunostimulation. A 1970s randomised study suggested that postoperative BCG, with or without 5-fluorouracil, could prolong disease-free interval in high-risk disease, which fueled multiple follow-on regimens and vaccine concepts [22].

However, larger cooperative-group experiences failed to confirm a meaningful cancer-specific survival benefit for BCG-based approaches. In National Surgical Adjuvant Breast and Bowel Project C‑01 (*n* >1,000), BCG produced at most modest signals that did not translate into improved colon-cancer–specific survival, while chemotherapy drove the clinically relevant benefit [23]. Similarly, an Eastern Cooperative Oncology Group trial of an irradiated autologous tumour-cell vaccine with BCG after resection did not improve long-term disease-free or overall survival [24]. A more nuanced result emerged with OncoVAX^®^ (autologous tumour cells + BCG), which reduced recurrence mainly in stage II rather than stage III disease, implying that if BCG-adjuvanted vaccination has a colorectal niche, it is likely earlier-stage, minimal-residual-disease settings with intact immune competence [25]. Overall, the colorectal experience supports using BCG as an adjuvant only when antigen strategy, stage selection and immune monitoring are optimised, rather than as a stand-alone stimulant.

## Why lung and colorectal programs rarely translated

Across lung and colorectal studies, several recurring factors likely limited translation into durable survival gains: delivery constraints, because most lung tumours were not injectable and postoperative exposure was variable; patient selection that mixed minimal residual disease with bulky disease, diluting any stage-restricted benefit; heterogeneous regimens and endpoints that were not aligned with immune kinetics or maintenance-like exposure; limited biomarker-driven enrichment and immune monitoring; and toxicity and dosing constraints that curtailed sustained immunologic pressure.

## BCG in HCC: preclinical ‘Heating’ via trained immunity and IFN-γ, supporting biomarker-guided in situ strategies

HCC is typically immunosuppressive (‘cold’), making it a plausible target for *in situ* immune ‘heating.’ In murine HCC models, systemic BCG vaccination inhibited established tumours, increased intratumoural CD4⁺/CD8⁺ T cells and M1-like macrophages, and engaged IFN‑γ–linked programs; in that setting BCG outperformed anti‑PD‑1 therapy [26]. Mechanistically, the effect appears to depend on trained-immunity pathways and IFN-γ signaling. When these were blocked, benefit diminished, supporting the idea that BCG is acting as a programmable innate primer rather than a nonspecific irritant [26]. From a translational standpoint, the most credible clinical path is not systemic BCG monotherapy but *in situ* strategies (intratumoural injection, peri-ablation or in combination with locoregional therapy) paired with biomarker-rich immune readouts [27].

### BCG in other cancers

Beyond bladder cancer, BCG applications remain mostly investigational and often preclinical, with clinical use limited by tumour accessibility, safety and inconsistent efficacy.

### Breast cancer

Breast cancer programs largely use BCG as an adjuvant within autologous or whole-cell vaccine platforms. In a small clinical study, an autologous tumour lysate/BCG approach was feasible and safe as an adjunct to standard therapy, though the evidence base remains thin by modern standards [28]. Building on this line of work, the platform has been further developed and formalised as ConvitVax, an autologous tumour cell vaccine combined with BCG and formalin, which has demonstrated acceptable toxicity and immune activation in preclinical and translational studies, including potential synergy with anti-PD-1 therapy in experimental models. This vaccine is expected to enter early-phase clinical testing in the near future [29, 30]. ConvitVax is now being evaluated clinically in metastatic breast cancer (NCT06023277). The practical limitation is delivery and safety: systemic BCG toxicity and the relative inaccessibility of many breast lesions make intratumoural strategies harder to scale than in skin or bladder [31].

### Sarcomas

Case-level signals have been reported in some sarcomas and hematologic malignancies, but they are too anecdotal to support routine use; they mainly reinforce that BCG’s effects are context-dependent and delivery-limited. For example, isolated partial responses have been described in rare sarcoma cases treated with intralesional BCG-based regimens, and veterinary experience (e.g., equine sarcoids) supports the concept that localised BCG can trigger regression in some mesenchymal tumours [31].

### Head and neck cancers

In head and neck cancers, the most consistent data are preclinical: BCG-conditioned immune stimulation can increase inflammatory cytokines, enhance antigen presentation and suppress tumour-cell proliferation and angiogenic signaling *in vitro* [32]. Reported effects include increased Th1-associated cytokines and upregulation of antigen-presentation machinery (e.g., (Human Leukocyte Antigen/Major Histocompatibility Complex class I) class I), which can improve immune recognition in experimental systems [33, 34]. While intriguing, these models do not resolve real-world safety constraints for intratumoural BCG in complex anatomic sites, so clinical translation remains speculative. Prior attempts at local delivery have been limited in part by concern for infectious complications and the practical difficulty of safely injecting tumours in high-risk head-and-neck locations [31].

### Hematologic malignancies

Evidence in hematologic malignancies is largely historical and inconsistent; any potential role would likely be as an immune-reconstitution adjunct after cytotoxic therapy rather than as a primary antitumour agent, and clinical translation remains uncertain. In preclinical leukemia models, post-chemotherapy BCG has been reported to improve survival in association with enhanced marrow recovery and innate immune activation, suggesting a plausible adjunctive niche that remains unproven clinically [31].

**Figure 1.**
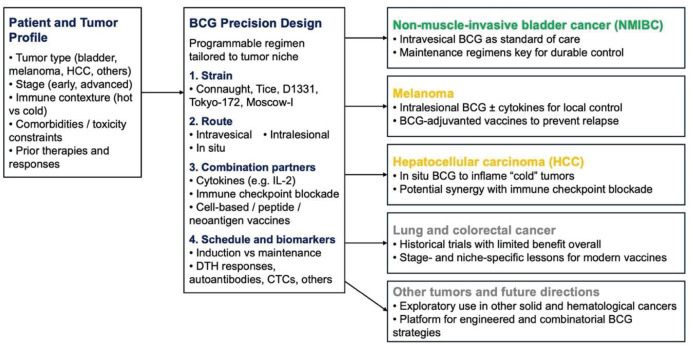
BCG in the fight against cancer: applications across tumour types.

Overall, BCG reliably stimulates immunity across tumour types, yet delivery barriers, systemic toxicity and uneven efficacy constrain its use outside bladder cancer. In practice, any progress beyond bladder cancer will likely require highly selected settings where local delivery is feasible and immune readouts can guide dosing and safety. Rational combinations and biomarker-guided strategies may expand its role, as summarised in Figure 1.

## Conclusion

This review traces BCG’s evolution from a century-old tuberculosis vaccine to a versatile oncologic tool. Its mechanisms span direct cytotoxicity, trained innate immunity and durable adaptive responses, while clinically meaningful differences among sub-strains influence outcomes. BCG remains definitive in NMIBC, shows renewed promise in melanoma -particularly within IL-2 combinations and cell-based vaccines and yields mixed or stage-limited signals in lung- colorectal and other tumours. Preclinical advances in HCC highlight BCG’s capacity to convert ‘cold’ tumours into immunologically ‘hot’ targets and, in models, to outperform checkpoint blockade when applied appropriately. Looking ahead, progress will depend on precise mechanism-guided-guided use rather than broad stimulation, anchored by three priorities: (1) strain selection informed by comparative genomics (i.e., ‘early’ versus ‘late’ sub-strains with differing immunogenic profiles) and tailored to tumour context; (2) leveraging trained immunity, as BCG induces epigenetic reprogramming of innate cells that may potentiate subsequent immunotherapies and (3) predictive biomarkers, including autoantibody signatures and immune-reactivity measures such as delayed-type hypersensitivity, to enrich for patients most likely to benefit and to guide dosing and safety monitoring.

## Conflicts of interest

The author(s) declare that they have no conflicts of interest.

## Figures and Tables

**Table 1.** Evidence chart of BCG applications beyond bladder cancer (representative evidence).

Tumor type	Delivery route	Best evidence	Signal	Practical niche	Key limitation
NMIBC	Intravesical	Multiple RCTs	Positive (standard)	Recurrence prevention; maintenance	Toxicity; supply; strain variability
Melanoma (cutaneous/in-transit)	Intralesional (± IL-2); adjuvant	Series/phase II; vaccine RCTs	Mixed to positive (local)	Injectable lesions; priming; select vaccines	Combination toxicity; accessibility limits
Lung cancer	Postoperative intrapleural; adjuvant	RCTs; phase III vaccine	Mostly negative	No established niche	Delivery barrier; nonreproducible benefit
Colorectal cancer	Postoperative adjuvant; vaccines	Cooperative-group RCTs; vaccine trials	Mostly negative	Possible MRD/vaccine niche	Heterogeneity; endpoint mismatch; small effects
HCC	Systemic vaccination; *in situ* priming	Preclinical (murine)	Promising (models)	Early-phase, mechanism-driven combos	Translation risk; safety in cirrhosis
Breast cancer	Vaccine adjuvant (autologous or whole-cell)	Small clinical studies	Feasible; immunogenic	Adjunct within vaccine programs	Limited validation
Head and neck cancers	Immune conditioning	Preclinical/*in vitro*	Preclinical signal	Early translational hypothesis	Lack of clinical trials
Sarcomas / hematologic	Varied; historical/anecdotal	Case-level/historical	Insufficient	None established	Evidence too sparse
